# Molecular diagnostic assay for pre-harvest detection of *Tilletia indica* infection in wheat plants

**DOI:** 10.3389/fmicb.2023.1291000

**Published:** 2023-11-01

**Authors:** Prem Lal Kashyap, Sudheer Kumar, Ravi Shekhar Kumar, Anju Sharma, Annie Khanna, Shubham Raj, Poonam Jasrotia, Gyanendra Singh

**Affiliations:** ICAR-Indian Institute of Wheat and Barley Research (IIWBR), Karnal, India

**Keywords:** Diagnostics, GAPDH, Karnal bunt, real time PCR, *Tilletia*, Wheat

## Abstract

The current study describes a new diagnostic method for the rapid and accurate detection of *Tilletia indica*, the pathogen accountable for causing Karnal bunt (KB) disease in wheat. This method uses quantitative real-time polymerase chain reaction (qPCR) and a primer set derived from glyceraldehyde 3-phosphate dehydrogenase (GAPDH) gene of *T. indica* to identify the presence of the pathogen. The qPCR assay using this primer set was found highly sensitive, with a limit of detection (LOD) value of 4 pg of *T. indica* DNA. This level of sensitivity allows for the detection of the pathogen even in cases of different growth stages of wheat, where no visible symptoms of infection on the wheat plants can be seen by naked eyes. The study also validated the qPCR assay on ten different wheat cultivars. Overall, this study presents a valuable molecular tool for rapid, specific and sensitive detection of KB fungus in wheat host. This method has practical applications in disease management, screening of wheat genotypes against KB and can aid in the development of strategies to mitigate the impact of Karnal bunt disease on wheat production.

## Introduction

Wheat is recognized as the prime cereal crop globally due to its widespread cultivation and essential role in human nutrition (Singh et al., 2023a). Fungal diseases in wheat are responsible for substantial yield losses, accounting for approximately 22% of total losses (Kayim et al., 2022). Karnal bunt (KB), provoked by fungal pathogen *T. indica*, is a significant quarantine disease that influences global wheat cultivation (Kashyap et al., 2023). KB was first identified in 1931 and was primarily confined to North–West India during its early years. Over the following decades, KB was officially reported in several other countries, including Afghanistan, Iraq, Nepal, Pakistan (specifically in Punjab and North–West Frontier Provinces), and Iran. It also appeared in Mexico (Sonora, Sinaloa, and Baja California Sur), Brazil (Rio Grande do Sul), the USA (New Mexico, Arizona, Texas, and California), and eventually South Africa (Northern Cape Province).^1^ The reason for the rapid spread of KB is the wheat seed movement, especially during and after the Green Revolution. This movement likely facilitated the dissemination of KB from its initial localized region to various other countries (Bishnoi et al., 2020). KB fungus infects the ovaries of growing wheat heads, resulting in the conversion of seeds into a black powder of teliospores (Fuentes-Davila and Duran, 1986; Kashyap et al., 2018). This severely impacts the quality and marketability of the grain (Sharma et al., 1998; Singh and Gogoi, 2011). Detecting Karnal bunt in wheat is challenging because not all seeds on an ear of wheat are infected, and infected seeds do not appear different from healthy ones. Additionally, symptoms are often not visible until harvest (Brar et al., 2018). It is worth to mention that *T. indica* is capable of being transmitted through seeds, soil, and air, making it particularly challenging to control and manage (Kaur and Kaur, 2005; Carris et al., 2006; Kashyap et al., 2019; Bishnoi et al., 2020). Several disease management options are available, including prophylactic measures, fungicide sprays, and deploying partially resistant wheat cultivars (Emebiri et al., 2021; Kashyap et al., 2022a; Singh et al., 2023b). However, these measures may not be entirely effective, especially under high disease pressure (Goates and Jackson, 2006; Bala et al., 2022). It is pertinent to mention that KB disease is sporadic and tends to invade during the late stages of host plant growth. Moreover, a single infected kernel can contain over 100,000 teliospores which are buried in the soil and are well-protected from both physical and chemical adverse conditions (Fuentes-Dávila et al., 2018; Thapa et al., 2022). Teliospores can persist in the soil for a long duration (Smilanick et al., 1989; Babadoost et al., 2004), making complete eradication of the disease through cultural practices and crop rotation complicated. Further, the localized mode of KB infection adds complexity to its management compared to other systemic smut diseases in plants (Fuentes-Davila, 1996; Riccioni et al., 2008). Conspicuously, *T. indica* infects wheat crops at a partial level, often not reaching a threshold level where it leads to significant economic losses. As a result, the chemical measures are generally neither applied nor completely effective. Additionally, the intricate nature of *T. indica* infection process makes it challenging to manage KB disease using both cultural practices and fungicides (Emebiri et al., 2019). Chemical fungicides such as Carbendazim, Triadimefon, and Propiconazole, when applied as foliar sprays during flowering or late booting stage have been effective against KB disease. However, concerns about their economic and environmental impact persist (Duveiller and Mezzalama, 2009; Kashyap et al., 2018). The efficacy of seed treatment with fungicides, such as Chlorothalonil and Carboxin + Thiram, is limited because teliospores are protected by the pericarp of infected kernels (Kumar et al., 2021). To address the challenges associated with Karnal bunt management, there is a prerequisite of a precise, responsive, and quick tool to detect and recognize *T. indica* fungus in wheat during its growth. Furthermore, it is crucial to have effective molecular markers that can differentiate *T. indica* from other pathogens that affect the wheat phyllosphere under field conditions as well as for large screening of KB resistant genotypes.

Traditional methods for diagnosing *T. indica* infection in wheat crops rely on morphological identification of symptoms in seeds and spore load (teliospore) counts after harvesting (Sharma et al., 2022). These morphological methods are tedious, time-consuming, and may not be very precise (Gurjar et al., 2021; Iquebal et al., 2021). Moreover, several smut-causing fungi, including *T. controversa*, *T. tritici*, *T. laevis*, *T. barclayana*, *T. ehrhartae*, and *T. walkeri*, share morphological similarities with *T. indica* and results in the misidentification when relying solely on the morphology or microscopic examination (Ferreira et al., 1996; Pascoe et al., 2005). Other technique such as isozyme analysis has been reported for KB detection (Luster et al., 1998). The prime limitation of this assay is that it requires at least 10 days duration for spore germination. Additionally, it demands specialized knowledge and expertise to perform accurately (Kutilek et al., 2001). Currently, polymerase chain reaction technology is considered a promising alternative for diagnosing *T. indica*. It is simple, rapid, accurate, and sensitive, making it suitable for detecting and identifying plant pathogens. Many of the PCR assays target the internal transcribed spacer (ITS) region (Levy et al., 2001; Kashyap et al., 2016; Gurjar et al., 2017; Gupta et al., 2022; Ren et al., 2022a), which has limited variation between *T. indica* and its closest relative, *T. walkeri* that differ by one nucleotide only (Bonde et al., 1997; Levy et al., 2001; Tremblay et al., 2021). This can affect sensitivity, a critical factor when dealing with diseases like Karnal bunt. Some assays require teliospore germination before molecular analysis, which can slow down the diagnostic process (Tan et al., 2010). Additionally, many previously developed assays are low-throughput, and international protocols involve time-consuming processes like morphological observations and spore isolation (Valente et al., 2019). A series of DNA-based methods have attempted to distinguish teliospores of *Tilletia* species from seeds or soil samples, which serve as the primary source of pathogen spread (Eibel et al., 2005; Chen et al., 2016; Valente et al., 2023). In earlier studies, PCR based techniques such as RAPD primer-mediated asymmetric polymerase chain reaction (Yuan et al., 2009), sequence-characterized amplified region (Liu et al., 2009; Gao et al., 2010), repetitive element polymerase chain reaction (Vesna et al., 2011), inter-simple sequence repeat (Gao et al., 2011), amplified fragment length polymorphism (Zhang et al., 2012), and sequence characterized amplified region (Ren et al., 2022a; Xu et al., 2022) have been successfully demonstrated for the identification of *Tilletia* species. However, real time qPCR based assays are more sensitive, precise, reliable and time saving than conventional PCR method (Zhang et al., 2012; Fang and Ramasamy, 2015; Yao et al., 2019). Moreover, real-time PCR (qPCR) eliminates the need to run gels after the reaction, reducing the risk of cross-contamination. At present, there is limited information available regarding the qPCR based assay for the diagnosis of *T. indica* infection in wheat host under field conditions.

Selecting the right target region is crucial for detecting *T. indica* infection in plants. Since the whole genomes of *T. indica* are publicly available, researchers have the opportunity to identify new target loci for molecular detection. This can be achieved through bioinformatic analysis of *T. indica* genomes and comparing them with other pathogens causing wheat diseases. Pandey et al. (2018) conducted a study comparing highly virulent (TiK) and low virulent (TiP) isolates of *T. indica*. They found that glyceraldehyde 3-phosphate dehydrogenase (GAPDH) plays a crucial role in determining virulence in *T. indica*. GAPDH is highly abundant in *T. indica* mycelium and serves as a virulence factor, similar to its role in other pathogenic fungi. Interestingly, GAPDH is not only present in the cytosol but also on the surface of virulent *T. indica* isolates. This surface presence of GAPDH contributes to fungal incursion and colonization of wheat tissues.

Real-time PCR has become a significant tool for the diagnosis and detection of phytopathogenic fungi over the last few decades (Kumar et al., 2013; Kashyap et al., 2020a). It has several advantages over conventional PCR methods in terms of sensitivity, reducing the peril of pseudo-positive results, and enabling quantitative analysis (Schena et al., 2006; Choudhary et al., 2020). Abdullah et al. (2018) reported real time PCR assay that concurrently differentiate and enumerate co-infections by *Parastagonospora nodorum* and *Pyrenophora tritici-repentis* in wheat. Loop-mediated isothermal amplification (LAMP) method has been reported for the diagnosis of *Tilletia* spp. (Gao et al., 2016; Pieczul et al., 2018). There are several studies that reported lower sensitivity of LAMP assay than qPCR (Ou et al., 2012; Yang et al., 2013; Nixon et al., 2014; Naveen and Bhat, 2020). Khan et al. (2018) also mentioned that LAMP assay was less sensitive than nested PCR and qPCR in diagnosis *Alternaria solani*. Similarly, qPCR assay developed by Frederick et al. (2000) was reported to detect 5 pg of DNA, showing relatively more sensitivity than LAMP assays by Gao et al. (2016) at ≥10 pg of DNA and Tan et al. (2016) at 10 pg of fungal DNA. Harper et al. (2010) highlighted that LAMP and real-time assays are highly specific, however, qPCR showed greater sensitivity for rapid detection of *Xylella fastidiosa* than LAMP assay. The most common drawback of the LAMP assay is the misamplifications that occur from redundant secondary structures. Even with the careful primer design and the availability of programs that confirm dimer and hairpin structure formation, there is no assurance that these structures will not be produced practically (Alhamid et al., 2023). Recently, droplet digital PCR (ddPCR) technique has been explored for the precise and sensitive diagnosis of *Tilletia caries* fungus in wheat (Ren et al., 2022b). ddPCR’s ability to provide highly sensitive and specific absolute quantification, combined with its reproducibility and tolerance to inhibitors, makes it a valuable emerging tools in the field of diagnostics (Xu et al., 2020). However, the prime demerits and concerns associated with ddPCR include high cost, technical complexity and a reduced dynamic range in comparison to qPCR (Jones et al., 2016). Another major concern with ddPCR is false-positive partitions in no template control (NTC) well (Kojabad et al., 2021). Most importantly, a droplet digital PCR system consists of multiple instruments (droplet generator, thermocycler, and droplet reader) that take up valuable lab space and require trained personnel for operation. Keeping aforementioned points in mind, the current study was planned for developing qPCR assay to differentiate and quantify *T. indica* in wheat under field conditions and for screening wheat genotypes against KB. To achieve this, research has been performed to design species specific oligonucleotide primers derived from the GAPDH gene and conducted validation tests to assess the performance of these primers in detecting *T. indica* in pure cultures as well as in artificially inoculated wheat seedlings at different growth stages of wheat under field conditions.

## Materials and methods

### Fungal isolates and DNA extraction

The study involves a total of 71 fungal isolates. A detailed description pertaining to host, pathogen identification, geographical region, year of isolation of the fungal isolates used in the current study is mentioned in Table 1. Usually, an annual crop health field survey is conducted every year to assess the wheat crop situation in the country. During the surveys at farmer’s field and grain mandies from 2017 to 2020, diseased samples (either plant parts or harvested seed) were collected. Among them, 50 were isolates of *T. indica*, and the remaining 21 isolates were from other unrelated fungal pathogens, majority of them were potential wheat pathogens (Table 1). These pathogens include *Tilletia caries*, *Blumeria graminis*, *Bipolaris sorokiniana*, *Alternaria alternata*, *Alternaria triticina*, *Fusarium graminearum*, *P. striiformis*, *Sclerotium rolfsii*, *P. triticina*, *Pyrenophora tritici*-*repentis*, *Rhizoctonia solani*, *Urocystis tritici*, *Ustilago tritici*, and *Ustilago hordei*. Ungerminated teliospores of *U. agropyri*, *Tilletia indica*, *Tilletia caries*, *Ustilago tritici*, and *Ustilago nuda* f. sp. *hordei* collected from various regions were directly processed for genomic DNA isolation. The mycelia of fungal isolates (*Fusarium graminearum*, *Alternaria triticina*, *Bipolaris sorokiniana*, *Pyrenophora tritici-repentis*, *Alternaria alternata*, *Sclerotium rolfsii*, and *Rhizoctonia solani*) were cultivated on potato dextrose broth (Hi Media, India) following single spore methodology for 7 days at 25 ± 2°C. The mycelial mat of each isolate was separated through sterile Whatman filter paper and ground to fine powder with mortar and pestle by incorporating liquid nitrogen. Total genomic DNA from these fungal isolates was extracted by following a protocol reported by Kumar et al. (2013). The purity and concentration of the isolated genomic DNA were assessed using a ScanDrop^2^ instrument from Analytik Jena, Germany. The isolated DNA was stored at a temperature of −20°C for future use.

**TABLE 1:** Fungal isolates used in the current study.

S. No.	Isolate code	Disease/host	NCBI accession No.	Geographical region	Isolation year	Source/references
1	*T. indica* KTi-19-1	Karnal bunt/wheat	MT503509	Punjab, India	2019	Kashyap et al., 2023
2	*T. indica* KTi-19-2	Karnal bunt/wheat	MT503510	Punjab, India	2019	Kashyap et al., 2023
3	*T. indica* KTi-19-3	Karnal bunt/wheat	MT503511	Punjab, India	2019	Kashyap et al., 2023
4	*T. indica* KTi-19-4	Karnal bunt/wheat	MT503512	Punjab, India	2019	Kashyap et al., 2023
5	*T. indica* KTi-19-5	Karnal bunt/wheat	MT503513	Punjab, India	2019	Kashyap et al., 2023
6	*T. indica* KTi-19-6	Karnal bunt/wheat	MT503514	Rajasthan, India	2019	Kashyap et al., 2023
7	*T. indica* KTi-19-7	Karnal bunt/wheat	MT503515	Uttarakhand, India	2019	Kashyap et al., 2023
8.	*T. indica* KTi-19-8	Karnal bunt/wheat	MT503516	Uttar Pradesh, India	2019	Kashyap et al., 2023
9	*T. indica* KTi-19-9	Karnal bunt/wheat	MT503517	Himachal Pradesh	2019	Kashyap et al., 2023
10	*T. indica* KTi-19-10	Karnal bunt/wheat	MT503518	Rajasthan, India	2019	Kashyap et al., 2023
11	*T. indica* KTi-19-11	Karnal bunt/wheat	MT503519	Rajasthan, India	2019	Kashyap et al., 2023
12	*T. indica* KTi-19-12	Karnal bunt/wheat	MT503520	Jammu, India	2019	Kashyap et al., 2023
13	*T. indica* KTi-19-13	Karnal bunt/wheat	MT503521	Punjab, India	2019	Kashyap et al., 2023
14	*T. indica* KTi-19-14	Karnal bunt/wheat	MT503522	Rajasthan, India	2019	Kashyap et al., 2023
15	*T. indica* KTi-19-15	Karnal bunt/wheat	MT503523	Haryana, India	2019	Kashyap et al., 2023
16	*T. indica* KTi-19-16	Karnal bunt/wheat	MT503524	Jammu, India	2019	Kashyap et al., 2023
17	*T. indica* KTi-19-17	Karnal bunt/wheat	MT503525	Punjab, India	2019	Kashyap et al., 2023
18	*T. indica* KTi-19-18	Karnal bunt/wheat	MT503526	Haryana, India	2019	Kashyap et al., 2023
19	*T. indica* KTi-19-19	Karnal bunt/wheat	MT503527	Uttarakhand, India	2019	Kashyap et al., 2023
20	*T. indica* KTi-19-20	Karnal bunt/wheat	MT503528	Jammu, India	2019	Kashyap et al., 2023
21	*T. indica* KTi-19-21	Karnal bunt/wheat	MT503529	Punjab, India	2019	Kashyap et al., 2023
22	*T. indica* KTi-19-22	Karnal bunt/wheat	MT503530	Punjab, India	2019	Kashyap et al., 2023
23	*T. indica* KTi-19-23	Karnal bunt/wheat	MT503531	Uttarakhand, India	2019	Kashyap et al., 2023
24	*T. indica* KTi-19-24	Karnal bunt/wheat	MT503532	Haryana, India	2019	Kashyap et al., 2023
25	*T. indica* KTi-19-25	Karnal bunt/wheat	MT503533	Punjab, India	2019	Kashyap et al., 2023
26	*T. indica* KTi-19-26	Karnal bunt/wheat	MT503534	Punjab, India	2019	Kashyap et al., 2023
27	*T. indica* KTi-19-27	Karnal bunt/wheat	MT503535	Punjab, India	2019	Kashyap et al., 2023
28	*T. indica* KTi-19-28	Karnal bunt/wheat	MT503536	Uttar Pradesh, India	2019	Kashyap et al., 2023
29	*T. indica* KTi-19-29	Karnal bunt/wheat	MT503537	Haryana, India	2019	Kashyap et al., 2023
30	*T. indica* KTi-19-30	Karnal bunt/wheat	MT503538	Uttar Pradesh, India	2019	Kashyap et al., 2023
31	*T. indica* KTi-19-31	Karnal bunt/wheat	MT503539	Uttar Pradesh, India	2019	Kashyap et al., 2023
32	*T. indica* KTi-19-32	Karnal bunt/wheat	MT503540	Rajasthan, India	2019	Kashyap et al., 2023
33	*T. indica* KTi-19-33	Karnal bunt/wheat	MT503541	Haryana, India	2019	Kashyap et al., 2023
34	*T. indica* KTi-19-34	Karnal bunt/wheat	MT503542	Punjab, India	2019	Kashyap et al., 2023
35	*T. indica* KTi-19-35	Karnal bunt/wheat	MT503543	Rajasthan, India	2019	Kashyap et al., 2023
36	*T. indica* KTi-19-36	Karnal bunt/wheat	MT503544	Punjab, India	2019	Kashyap et al., 2023
37	*T. indica* KTi-19-37	Karnal bunt/wheat	MT503545	Rajasthan, India	2019	Kashyap et al., 2023
38	*T. indica* KTi-19-38	Karnal bunt/wheat	MT503546	Himachal Pradesh	2019	Kashyap et al., 2023
39	*T. indica* KTi-19-39	Karnal bunt/wheat	MT503547	Himachal Pradesh	2019	Kashyap et al., 2023
40	*T. indica* KTi-19-40	Karnal bunt/wheat	MT503548	Rajasthan, India	2019	Kashyap et al., 2023
41	*T. indica* KTi-19-41	Karnal bunt/wheat	MT503549	Rajasthan, India	2019	Kashyap et al., 2023
42	*T. indica* KTi-19-42	Karnal bunt/wheat	MT503550	Uttar Pradesh, India	2019	Kashyap et al., 2023
43	*T. indica* KTi-19-43	Karnal bunt/wheat	MT503551	Uttar Pradesh, India	2019	Kashyap et al., 2023
44	*T. indica* KTi-19-44	Karnal bunt/wheat	MT503552	Jammu, India	2019	Kashyap et al., 2023
45	*T. indica* KTi-19-45	Karnal bunt/wheat	MT503553	Jammu, India	2019	Kashyap et al., 2023
46	*T. indica* KTi-19-46	Karnal bunt/wheat	MT503554	Jammu, India	2019	Kashyap et al., 2023
47	*T. indica* KTi-19-47	Karnal bunt/wheat	MT503555	Himachal Pradesh, India	2019	Kashyap et al., 2023
48	*T. indica* KTi-19-48	Karnal bunt/wheat	MT503556	Himachal Pradesh, India	2019	Kashyap et al., 2023
49	*T. indica* KTi-19-49	Karnal bunt/wheat	MT503557	Haryana, India	2019	Kashyap et al., 2023
50	*T. indica* KTi-19-50	Karnal bunt/wheat	MT503558	Himachal Pradesh, India	2019	Kashyap et al., 2023
51	*T. caries* WHB1	Hill bunt/wheat	OR452911	Uttarakhand, India	2019	This study
52	*T. caries* WHB5	Hill bunt/wheat	OR452914	Himachal Pradesh, India	2019	This study
53	*Bipolaris sorokiniana* WLB-19-176	Spot blotch/wheat	OM022942	West Bengal, India	2019	Kashyap et al., 2022b
54	*B. sorokiniana* WLB-19-118	Spot blotch/wheat	OM037077	Himachal Pradesh, India	2019	Kashyap et al., 2022b
55	*B. sorokiniana* WLB-19-110	Spot blotch/wheat	OM037085	Uttarakhand, India	2019	Kashyap et al., 2022b
56	*B. sorokiniana* WLB-19-52	Spot blotch/wheat	OM023811	Karnataka, India	2019	Kashyap et al., 2022b
57	*Puccinia striiformis* f.sp. *tritici* race 238S119	Yellow rust/wheat	–	Bilaspur, Himachal Pradesh	2014	Flowerdale, Shimla
58	*Puccinia striiformis* f.sp. *tritici* race 110S119	Yellow rust/wheat	–	Ropar, Punjab	2014	Flowerdale, Shimla
59	*Fusarium graminearum* NFG1	Head scab/wheat	ON215826	Punjab, India	2018	Kaul et al., 2022
60	*Fusarium graminearum* NFG25	Head scab/wheat	ON215850	Haryana, India	2019	Kaul et al., 2022
61	*Pyrenophora tritici*-*repentis*	Tan spot/wheat	OK666833	Haryana, India	2020	This study
62	*Ustilago tritici* WLS17-PUN-1	Loose smut/heat	ON127421	Punjab, India	2017	This study
63	*Urocystis agropyri* FLS1	Flag smut/wheat	MG386989	Uttarakhand, India	2015	Kashyap et al., 2020b
64	*Alternaria alternata* WBPA05	Black point/wheat	OR457685	Haryana, India	2019	This study
65	*Alternaria triticina* WBPAA1	Black point/wheat	OR457684	Haryana, India	2019	This study
66	*Puccinia triticina* race77-5	Leaf rust/wheat	–	Tamil Nadu, India	1992	Flowerdale, Shimla
67	*Ustilago hordei* HP-256	Covered smut/barley	OR452997	Himachal Pradesh, India	2016	This study
68	*Sclerotium rolfsii* SR-1	Foot rot/wheat	OR457686	Karnataka, India	2018	This study
69	*Rhizoctonia solani* RRS-4	Sheath blight/rice	–	Haryana/India	2018	This study
70	*Blumeria graminis* f. sp. *tritici* BGTHP26	Powdery mildew/wheat	MT462292	Himachal Pradesh, India	2019	This study
71	*Blumeria graminis* f. sp. *tritici* BGTHR7	Powdery mildew/Wheat	MT462304	Haryana, India	2019	This study

### Development of *T. indica* specific primers

**FIGURE 1:**
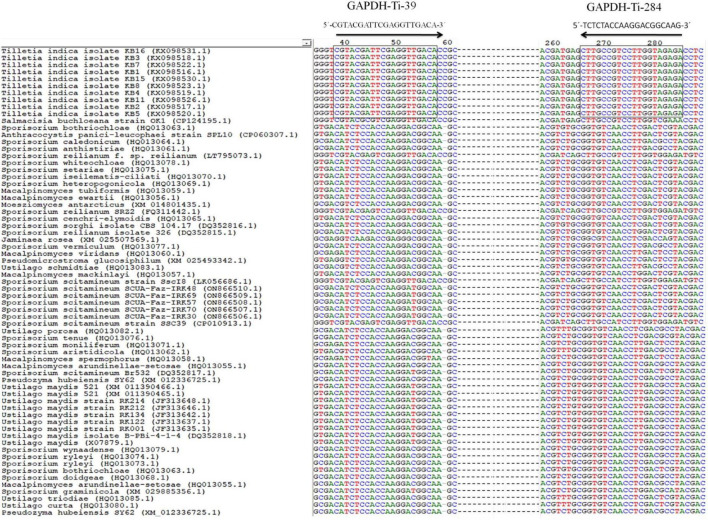
Alignment of GAPDH gene sequences from *T. indica* showing the position and orientation of unique region of *T. indica*. The GAPDH-Ti-39 (Forward) and GAPDH-Ti-284 (reverse) primers used for the PCR and qPCR assay that are identical between the species are shown. Consensus nucleotides are indicated with a rectangle box.

For designing the species-specific primers for *T. indica*, GAPDH gene sequences of *T. indica* from the NCBI GenBank were downloaded. Clustal W software (Thompson et al., 1997) was used to align multiple sequences (Figure 1). Sequence alignment was performed to identify conserved regions and polymorphic sites, which later used to design specific primers. Using the Primer 3 plus software,^2^ species-specific primers (Table 2) were designed to amplify only the target species, i.e., *Tilletia indica* and not other related organisms. Basic Local Alignment Search Tool (BLAST) tool^3^ was used to corroborate the specificity of identified primers and to ensure that the primers did not amplify similar sequences in other microorganisms present in the NCBI GenBank database.^4^ The primer quality was assessed using the IDT oligo analyzer.^5^ This involves checking for potential issues like secondary structure formation, self-complementarity, and dimer formation, as these can affect the efficiency and specificity of PCR reactions.

**TABLE 2:** Primer developed in the current study.

NCBI accession no.	Primer name	Sequence (5′-3′)	Amplicon size (bp)	Tm (°C)
KX098531	GAPDH-Ti-39	CGTACGATTCGAGGTTGACA	243	55
	GAPDH-Ti-284	TCTCTACCAAGGACGGCAAG		

### PCR amplification assay

Polymerase chain reaction (PCR) experiment for the amplification of a DNA target using specific primers was performed. The total reaction volume was 10 μl and encompassed 1 μl of template DNA with a concentration of 40 ng μl^–1^. A total

of 5 μl of GoTaq Green master mix by Promega Biotech, India was used. A total of 1 μl of each forward and reverse primer was added, and these primers had a concentration of 10 μM. Nuclease-free water was incorporated to make up the remaining volume to 10 μl. A control check without genomic DNA (NC) was also included. The thermal cycler used was the Sure Cycler 8800 (Agilent Technologies). The PCR program composed of initial denaturation at 94°C for 5 min followed by 35 cycles of: denaturation at 94°C for 60 s, annealing at 55°C for 60 s and extension at 72°C for 60 s. A final extension step at 72°C for 10 min was maintained with holding time at 4°C. The results of PCR amplicon generation were analyzed through electrophoresis. A 1.2% agarose gel in 1 × TBE buffer was used for this purpose. Electrophoresis was run for 1 h at 90 V. To estimate the size of the amplicon (the DNA fragment that was amplified), a 100 bp DNA ladder by Promega Biotech India Pvt. Ltd., was used as a reference.

### *In vivo* validation of the assay

The field experiment was performed at ICAR-Indian Institute of Wheat and Barley Research (IIWBR) in Karnal, India (29°42′19.5″N 76°59′24.8″E), during the *rabi* cropping season of 2020–2021. Two wheat cultivars, PBW343 and HD2967, were used in the experiment for preliminary validation of the developed markers under field conditions. The soil texture of experimental field was sandy loam (62.1% sand, 26.9% silt, and 11.0% clay) with pH 7.8 and electrical conductivity 0.25 dS/m. The soil was having 0.42% organic carbon, 193 Kg ha^–1^ available N, 17.9 Kg ha^–1^ available P, and 241 Kg ha^–1^ available K. Plantation of the viable (95% seed germination) foundation wheat seeds (99% seed purity) was done in experimental plot (2 m^2^) with 1 m long rows with a space of 22 cm between rows. Each cultivar was sown in three replicates. Sowing operations of the wheat seeds were performed in the second week of November, which is the optimal time for wheat sowing in North India. *T. indica* isolates (Table 1) were grown on Petri plates amended with potato dextrose agar (Hi Media, India) for 7 days. The load of the liquid suspension of secondary sporidia (5 × 10^6^ ml^–1^) in sterilized distilled water was optimized with the help of a hemocytometer. During evening hours, two milliliter of standardized liquid suspension was injected into tillers of each wheat cultivar at Zadok’s growth stage 49 (ZGS49; boot leaf stage) using a hypodermal syringe (Aujla et al., 1989). High humidity (>70%) was sustained by regular mist sprays. At the stage of crop maturity, ears heads of the inoculated tillers were harvested. Each seed from the inoculated tillers was inspected for the presence of KB teliospores. The severity of infection was recorded using numerical values (0, 0.25, 0.50, 0.75, and 1.0) to indicate infection grade. The percent coefficient of infection (CI) was calculated using a specific formula mentioned by Kashyap et al. (2017). Genomic DNA was extracted from healthy and *T. indica*-inoculated wheat spikes at various growth stages [ZGS 59 (ear emergence stage), ZGS 69 (flowering stage), ZGS77 (milking stage) ZGS87 (dough stage) and ZGS92 (ripening stage)]. PCR assays were performed using species-specific primers, and gel electrophoresis by employing the procedure mentioned earlier.

### Real-time PCR assay and melting curve analysis

A quantitative real-time polymerase chain reaction (qRT-PCR) was performed by employing SYBR Green-I dye (Thermo Scientific, USA) on the AriaMx Real-Time PCR machine (Agilent Technologies, USA). The qPCR cocktail was set-up by combining the following components in a 20 μl reaction volume: 10 μl SYBR Green qPCR master mix (Thermo Scientific, USA), 10 picomoles of each primer (forward and reverse) and 1 μl of genomic DNA, with a concentration ranged from 40 to 0.004 ng μl^–1^. qPCR cycling conditions included initial heat activation at 94°C for 5 min followed by amplification with 35 cycles using a 2-step cycling method. This included denaturation at 94°C for 30 s and annealing at 55°C for 60 s. Melting curve analysis was performed after the amplification cycles to check the specificity of the PCR product. The plate was heated to 95°C for 30 s to denature the DNA. It is then incubated at 65°C for 30 s. Finally, it was heated again to 95°C for 30 s. Fluorescence is measured once per cycle at the completion of the extension step. The machine automatically computed the cycle quantification (Cq) values and were used to estimate the initial amount of target genomic DNA in the sample.

### Standard curves construction for absolute quantification of *T. indica*

An experiment was performed for absolute quantification of *T. indica* by constructing a standard curve for the GAPDH-Ti-39/GAPDH-Ti-284 primer. Genomic DNA was serially diluted with nuclease-free water to create a range of concentrations from 40 to 0.004 ng reaction^–1^. Each concentration was tested in three replicates independently. The entire experiment performed twice in an independent manner for confirming the consistency of results and ruling out any experimental errors. A no template control (NC) was also used to ensure the specificity of the reaction and avoid cross contamination.

### qPCR based detection of *T. indica* fungus load in wheat cultivars

Ten different wheat cultivars (DBW173, DBW187, DBW303, HD2967, HD3086, DBW252, PBW343, RAJ4083, WH542, and WL711) were used in the experiment to validate the applicability of the developed assay to check the genotype influence at wheat KB susceptible stage, i.e., boot leaf stage (ZGS-49 stage). Healthy seeds of these wheat cultivars were planted and grown under field conditions during the *rabi* season of 2021–2022. At the boot leaf stage (ZGS-49 stage), the wheat plants were artificially inoculated with *T. indica* at a concentration of 5 × 10^6^ sporidia ml^–1^. Wheat samples from each cultivar (1 gram each) were harvested at ZGS-77. Total genomic DNA was extracted from the collected wheat samples. The methodology used for DNA isolation was similar to a previous section. Real-time PCR assays were performed on the extracted genomic DNA. The qPCR cocktail (20 μl) was prepared by incorporating 10 μl SYBR Green qPCR master mix (Thermo Scientific, USA), 10 picomoles of each primer (forward and reverse) and 1 μl of genomic DNA. A negative control (NC) was included in the experiment. In this control, the DNA template was replaced by nuclease free water. qPCR cycling conditions were as follows: initial heat activation at 94°C for 5 min followed by amplification with 35 cycles using a 2-step cycling of denaturation at 94°C for 30 s and annealing at 55°C for 60 s. Melting curve analysis was performed after the amplification cycles to check the specificity of the PCR product. The plate was heated to 95°C for 30 s to denature the DNA. It is then incubated at 65°C for 30 s. Finally, it was heated again to 95°C for 30 s. Fluorescence is measured once per cycle at the end of extension step. Cycle quantification (Cq) values were used to estimate the initial amount of target genomic DNA in the sample. The assay was performed twice.

### Statistical analysis

The significance of the data generated for KB disease was statistically analyzed by conducting an analysis of variance (ANOVA). DMRT (Duncan’s Multiple Range Test) was carried out for *post hoc* comparative analysis of the mean data. Genetic analysis and sequence alignments were carried out using MEGA 10 software (Tamura et al., 2021). The *R*^2^ value was calculated according to the method described by Bustin et al. (2009). The criteria outlined by Broeders et al. (2014) were followed to determine the efficiency (which should be in the range of 90–110%), repeatability (with a relative standard deviation ≤25%), and linearity (with *R*^2^ ≥ 0.98) of the qPCR assay.

## Results

### Design of *T. indica*-specific primers

The result of the *in silico* analysis to monitor the homogeneity or genetic consistency of a specific genomic region (GAPDH) within isolates of *T. indica* was illustrated in Figure 1. Primers named GAPDH-Ti-39 and GAPDH-Ti-284 were designed on the basis of comparison of *T. indica* regions of divergence with other related species (Table 2). The amplification product generated by these primers had a size of 243 bp. The melting temperature of the DNA fragment was determined as 57°C. The BLAST analysis against the NCBI database was performed using BLAST 2.2.14.^6^ The designed primer pair was found specific for *T. indica* (Figure 1). Besides this, *in silico* results obtained after multiple alignments of GAPDH amino acid sequences of *T. indica* with animal and plant GAPDH amino acid sequences indicated that *T. indica* GAPDH region is unique and not matched with plant and animal GAPDH amino acid sequences (Supplementary Figure 1).

### Determination of primers specificity

**FIGURE 2:**
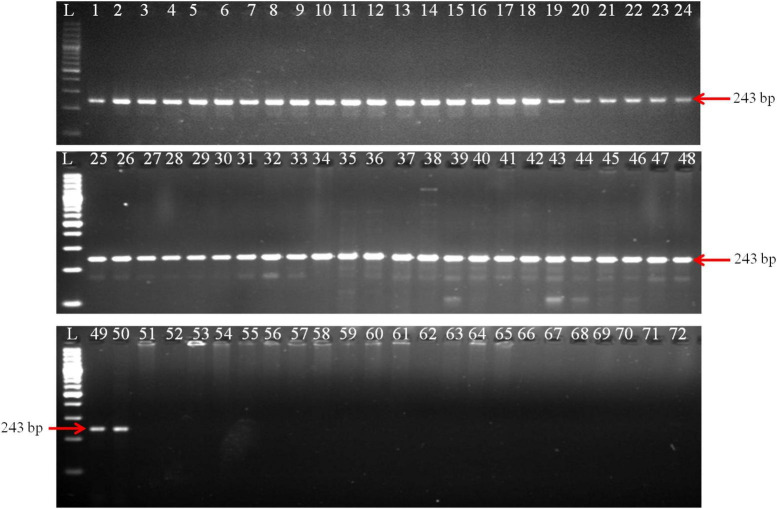
Polymerase chain reaction (PCR) amplification of GAPDH gene fragments from *T. indica* isolates using GAPDH-Ti-39 (Forward) and GAPDH-Ti-284 (reverse) primers. Lane 1–71 indicates the isolates as per Table 1. Lane72: NC (No template control); L: 100 bp DNA Ladder.

The PCR reaction successfully generated a 243 bp amplicon from all 50 isolates of *T. indica* (Figure 2). This suggests that the primer pair used in your PCR assay was specific to *T. indica* and that all the fifty isolates of *T. indica* share the same DNA sequence at the target region, resulting in the production of the same 243 bp product. For the remaining 21 isolates representing different fungal species, including *T. caries*, *B. graminis*, *B. sorokiniana*, *A. alternata*, *A. triticina*, *F. graminearum*, *P. striiformis*, *S. rolfsii*, *P. triticina*, *P. tritici*-*repentis*, *R. solani*, *U. tritici*, *U. agropyri*, and *U. hordei*, no amplified products were generated (Figure 2). This indicates that the primer pair used for PCR did not bind or amplify DNA from these fungal species.

### Detection of *T. indica* in infected plants at different growth stages

**FIGURE 3:**
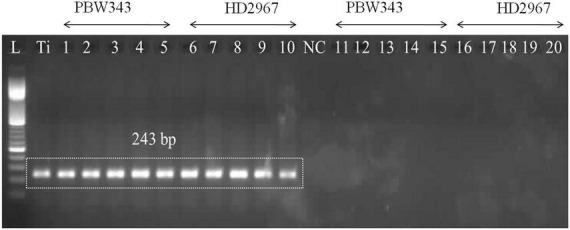
Polymerase chain reaction (PCR) amplification using DNA extracted from wheat seedlings of two different cultivars (PBW343 and HD2967) at five different crop stages, i.e., ZGS 59 (ear emergence stage), ZGS 69 (Flowering stage), ZGS77 (Milking stage) ZGS87 (Dough stage) and ZGS92 (ripening stage) using GAPDH-Ti-39/GAPDH-Ti-284 primer set. L: DNA ladder 100 bp; Ti, positive control (*T. indica* KTi-19-1); NC: Negative control (without KTi-19-1DNA template), 1–5: Zadoks growth stage DNA obtained from inoculated wheat seedlings at different growth stages; Lane 11–20: negative healthy control (DNA obtained from wheat seedlings, where only water injected in place of *T. indica* inoculation).

The PCR assay utilized the GAPDH-Ti-39/GAPDH-Ti-284 primer pair and successfully generated the specific amplicon (243 bp) from infected wheat varieties, namely PBW343 and HD2967. The specific amplicon was detected in infected wheat samples at all of these growth stages (i.e., ZGS 59, ZGS 69, ZGS 77, ZGS 87, and ZGS 92). Importantly, there was no amplification of the specific amplicon detected in healthy tissue from any of the growth stages (Figure 3). The observation pertaining to the occurrence of *T. indica* was made in the harvested seeds from the *T. indica*-inoculated wheat spikes (Table 3). During 2020–2021, the coefficient of infection (CI) were reported as 44.50% for PBW343 and 33.46% for HD2967 (Table 3), indicating the extent of disease infection in these wheat varieties.

**TABLE 3:** Coefficient of infection in artificially inoculated wheat varieties during 2020–2021.

Wheat variety	KB (%)
PBW343	44.50 ± 1.96^b^
HD2967	33.46 ± 0.97^a^

Means followed by the same letter are not significant different by the least significant difference test (*P* ≤ 0.05).

### Real-time PCR for absolute quantification of *T. indica*

**FIGURE 4:**
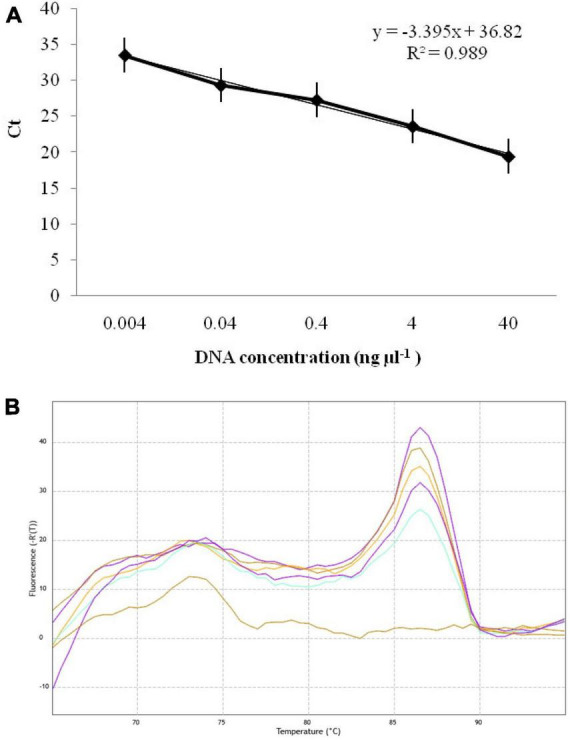
**(A)** Standard curves displaying the regression between DNA log quantities (ng, *X*-axis) and cycle thresholds (Ct, *y*-axis) for qPCR assay using GAPDH-Ti-39 (Forward) and GAPDH-Ti-284 (reverse) primers. The standard curve was developed with serial dilutions (40 to 0.004 ng μl^–1^) of *T. indica* KTi-19-1; **(B)** melting curve showing melting temperatures (86.50) required for 243 bp qPCR product amplification with GAPDH-Ti-39/GAPDH-Ti-284 primers.

The qPCR experiment using GAPDH-Ti-39/GAPDH-Ti-284 primers resulted in Ct (Cycle threshold) values ranging from 19.42 ± 0.03 to 33.53 ± 0.03 when analyzing different dilutions of *T. indica* DNA. The assay was able to detect *T. indica* DNA down to a concentration as low as 0.004 ng per microliter (Figure 4A). There is a linear correlation between the Ct values obtained in the qPCR assay and the concentration of the target *T. indica* DNA. The coefficient of determination (*R*^2^) was greater than 0.989, indicating a strong and positive correlation between the Ct values and DNA concentration. The linear equation *y* = −3.395x + 36.82 describes this relationship, where “y” represents Ct values, and “x” represents the target DNA concentration (Figure 4A). The melting temperature (Tm) for specific amplicon generation was determined to be 86.50 ± 0.50°C, indicating the specificity of the amplicon (Figure 4B).

### qPCR quantification of *T. indica* DNA in wheat varieties

The GAPDH-Ti-39 and GAPDH-Ti-284 primers were used in the qPCR analysis, and they produced distinct fluorescent signals from all the inoculated wheat samples. During 2021–2022, the mean Ct (Cycle threshold) values for *T. indica* inoculated wheat cultivars at ZGS87 (Dough stage) were measured and were ranged between 19.54 (cv. PBW343)–31.45 (cv. DBW187) (Table 4). The corresponding genomic concentration of *T. indica* in the wheat plant ranged from 0.001 (cv. DBW187) to 33.60 ng μl^–1^ (cv. PBW343). Maximum CI value was observed in cultivar PBW343 (44.50 ± 1.24%) followed by WL711 (38.62 ± 1.99%), HD2967 (35.46 ± 1.77%), WH542 (34.78 ± 1.45%), DBW303 (32.10 ± 1.65%), DBW173 (19.30 ± 0.58%), DBW252 (18.80 ± 1.32%), RAJ4083 (16.30 ± 0.95%), HD3086 (15.70 ± 1.24%), DBW187 (13.50 ± 0.42%) (Table 4). This suggests varying degrees of infection among the different wheat cultivars.

**TABLE 4:** Field evaluation of the specificity of primers to quantify *T. indica* load in artificially inoculated wheat varieties by qPCR assay during 2021–2022.

Wheat variety	KB (%)	Ct value	DNA concentration of *T. indica* (ng μ l^–1^)
DBW173	19.30 ± 0.58^c^	27.54 ± 1.2^f^	0.036 ± 0.001^e^
DBW187	13.50 ± 0.42^a^	31.45 ± 0.95^j^	0.001 ± 0.000^a^
DBW303	32.10 ± 1.65^d^	25.12 ± 0.25^e^	0.790 ± 0.12^e^
HD2967	35.46 ± 1.77^e^	23.25 ± 0.55^c^	1.480 ± 0.88^g^
HD3086	15.70 ± 1.24^b^	30.12 ± 0.26^i^	0.008 ± 0.002^c^
DBW252	18.80 ± 1.32^c^	28.45 ± 0.42^g^	0.020 ± 0.001^d^
PBW343	44.50 ± 1.24^g^	19.54 ± 0.52^a^	33.60 ± 0.04^i^
RAJ4083	16.30 ± 0.95^b^	29.45 ± 0.64^h^	0.002 ± 0.00^b^
WH542	34.78 ± 1.45^e^	24.51 ± 0.95^d^	1.020 ± 0.002^f^
WL711	38.62 ± 1.99^f^	21.45 ± 0.77^b^	9.110 ± 0.11^h^
NC	–	ND	–

Average of Ct values obtained from three replicates. NC, no template control; ND, not determined. Means followed by the same letter are not significant different by the least significant difference test (*P* = 0.05).

## Discussion

Karnal bunt is a significant quarantine fungus, and its detection in wheat crops is crucial for wheat researchers and implementing regulatory procedures related to global surveillance and quarantine (IPPC, 2016; Singh et al., 2023b). Traditional methods such as microscopic analysis, isozyme analysis, and pathogenicity tests have been used for seed testing and confirming the presence of *T. indica* (Kashyap et al., 2011; Iquebal et al., 2021). However, these methods have limitations, including subjectivity in observation and examination. Spore morphology analysis, in particular, is mentioned as being unable to distinguish *T. indica* from related *Tilletia* species and other wheat pathogens, leading to incorrect identifications (Ferreira et al., 1996; Smith et al., 1996; Pimentel et al., 1998; Castlebury and Carris, 1999). PCR methods have been developed for diagnosing various plant pathogens, including *T. indica*. These methods are favored in plant disease diagnostic laboratories due to their sensitivity, specificity, ease of execution, and rapid reporting (Kumar et al., 2013; Singh et al., 2014; Kashyap et al., 2016; Sharma et al., 2017; Chakdar et al., 2019; Kaur et al., 2020). Additionally, they require only a small amount of plant tissue for testing. At present, there is no established PCR-based diagnostic assay for confirming the presence of *T. indica* in different growth stages of wheat under field conditions. Such assays could be valuable for rapid field screening of wheat germplasm to identify Karnal bunt disease in breeding programs. In present research, efforts have been made to identify specific genomic regions that can be used to design *T. indica*-specific markers for accurate and precise detection of KB disease in wheat. These markers could potentially improve the accuracy and reliability of diagnostic assays for *T. indica*.

Previous research reports have explored the potential of using specific genetic regions, such as the internal transcribed spacer (ITS) and mitochondrial regions, to develop species-specific genetic markers (Levy et al., 2001; Tan and Murray, 2006; Thirumalaisamy et al., 2011; Gao et al., 2016; Kuzdraliński et al., 2017; Kashyap et al., 2020b; Gupta et al., 2022). However, the current study is the first of its kind to develop novel *T. indica*-specific markers that are derived from the GAPDH gene of *T. indica*. The purpose of these markers is to accurately identify the presence of *T. indica* infection in different wheat growth stages under field conditions.

The selection of an appropriate target region is a critical step in molecular detection of *T. indica* in wheat. In this study, GAPDH gene region was chosen for analysis because it is implicated in determining virulence in *T. indica*, making it a promising target (Pandey et al., 2018). It is worth to mention here that GAPDH exists in nearly all organisms (Henry et al., 2015). For instance, wheat has 22 GAPDH genes dispersed throughout the genome. As a result, it was decided to conduct bioinformatic analysis of the GAPDH region from various fungi, animals and wheat available in the NCBI database. This analysis likely involved comparing and identifying unique sequences or regions specific to *T. indica*. Multiple alignments of animal, fungi, wheat, and *T. indica* GAPDH amino acid sequences revealed that *T. indica GAPDH* identified for the development of species-specific marker was distinct and did not match with wheat, fungi, and animal amino acid sequences studied by Zeng et al. (2016). This clearly revealed that the GAPDH region used for the development of *T. indica* specific markers is unique and suitable for *T. indica* detection in wheat. As a consequence, *T. indica* specific primer set was developed by targeting GAPDH region that produces a single, specific DNA band of 243 bp during PCR assay. The designed primers were shown to be effective in generating the desired amplicons from the genomic DNA of various fungal pathogens responsible for diseases like head scab, yellow rust, powdery mildew, and others in wheat. Analogous report regarding the development of GAPDH based genetic markers for the detection of *Colletotrichum camelliae* fungus in tea host has been reported by He et al. (2020). A qPCR assay based on GAPDH gene with LOD of 80 fg μl^–1^ *C. kahawae* DNA in *Coffea arabica* has been demonstrated by Tao et al. (2013).

At present, for both culture dependent and PCR based procedures, the severity of infection and LOD (i.e., detection sensitivity) of each infected plants is still unclear in case of wheat-*T. indica* system. Therefore, a PCR and qPCR assay were reported in current study for species specific identification of *T. indica* from pure cultures as well as for the confirmation of *T. indica* presence in wheat plants at five different growth stages [i.e., ZGS 59 (ear emergence stage), ZGS 69 (flowering stage), ZGS77 (milking stage), ZGS87 (dough stage), and ZGS92 (ripening stage)] of two different cultivars. The study confirms that the developed assay was able to diagnose *T. indica* infection in all the growth stages of asymptomatic wheat tissues, which otherwise seen with naked eye only after crop harvesting. In terms of sensitivity, LOD of 1 pg of *T. indica* DNA in DBW187 has been recorded in present research by employing new primers in qPCR assay and found more sensitive than earlier reported qPCR assays in case of *T. indica* fungus (Frederick et al., 2000; Gao et al., 2016; Tan et al., 2016). A series of real-time PCR assays for detecting fungal species in plants have been developed by earlier workers. For instance, a qPCR assay targeting the *GAPDH* gene was reported by Ciampi-Guillardi et al. (2020) which have the potential to detect 3 pg of *S. sclerotiorum* DNA and 300 fg of both *C. truncatum* and *C. cassiicola* DNA in soybean seeds. Besides this, LOD of 4 pg of *C. cassiicola* DNA in soybean seeds (Guimarães et al., 2017) and 191.31 fg μl^–1^ of *Ilyonectria robusta* DNA in ginseng roots have been obtained using SYBR Green I based qPCR assay (Jiang et al., 2023). Similarly, a SYBR Green I real-time PCR assay for detecting *T. laevis* with a 100 fg μl^–1^ detection limit has also been reported by Xu et al. (2020). However, it is worth to mention that our results are comparable with the study of Tremblay et al. (2021), who reported LOD as low as 0.1 pg *T. indica* DNA in wheat seeds using qPCR method. Similarly, qPCR assay developed by Frederick et al. (2000) and Tan et al. (2016) was documented to detect as low as 5 and 10 pg of fungal DNA, respectively.

It is important to mention here that the detection limit of the qPCR assay was noticed to be 4 pg of total DNA isolated from pure fungal cultures of *T. indica*. This is more to the sensitivity of the LAMP assay for *T. controversa* with the LOD of 5 pg total DNA of pure fungal cultures (Sedaghatjoo et al., 2021). Similar reports of lower sensitivity of the LAMP assay for *T. indica* with the LOD of 10 pg reported by Gao et al. (2016) and Tan et al. (2016). However, it is less sensitive than the reported LOD of 0.001 pg for the LAMP assay not differentiating among *T. caries*, *T. controversa*, and *T. laevis* (Pieczul et al., 2018). Therefore, further comparative investigation with respect to qPCR, LAMP and ddPCR to determine the detection limit and their correlation with fungal biomass per wheat spike is highly warranted. But irrespective of this, the unambiguous results obtained in the present study by using qPCR to detect *T. indica* biomass in different wheat varieties advocate that it might play an essential role in the rapid and effective screening of genotypes against KB disease. Nevertheless, there is no specific DNA microarray available for the detection of fungal pathogens of wheat pathogens. The assay described here is an initial study of identifying GAPDH as a potential region for the identification of *T. indica*. Therefore, for developing large-scale and robust systems for screening wheat materials against KB, efforts should be made to explore, develop, and validate *T. indica*-specific probes targeting the entire genomes of *T. indica* to improve testing efficiency and reduce costs.

## Conclusion

The study presents a valuable molecular tool for rapid, specific and sensitive detection of KB fungus in wheat host. Identification and quantification of KB based on qPCR can be applied in the screening process for KB resistance in wheat as well as in certification and breeding processes at early stages of plant development. It could also assist the selection of potential resistance donors for breeding. However, there is room for further improvement through the exploration of combined post-harvest disease severity assessments and large-scale qPCR or GADPH based array, aiming to enhance sensitivity and optimization. Additionally, we accentuated the need for further investigation into the relationship between *T. indica* DNA concentrations and post-harvest disease severity across different locations and years to unlock the full potential of these new assays for high-throughput screening of wheat germplasm against KB.

## Data availability statement

The datasets presented in this study can be found in online repositories. The names of the repository/repositories and accession number(s) can be found in the article/Supplementary material.

## Author contributions

PK: Conceptualization, Funding acquisition, Investigation, Methodology, Project administration, Software, Validation, Writing – original draft, Writing – review and editing. SK: Funding acquisition, Investigation, Methodology, Project administration, Supervision, Validation, Visualization, Writing – review and editing. RK: Data curation, Investigation, Methodology, Validation, Visualization, Writing – review and editing. AS: Data curation, Formal analysis, Investigation, Methodology, Software, Validation, Visualization, Writing – review and editing. AK: Formal analysis, Investigation, Methodology, Software, Validation, Visualization, Writing – review and editing. SR: Investigation, Methodology, Validation, Writing – review and editing. PJ: Project administration, Resources, Software, Supervision, Writing – review and editing. GS: Funding acquisition, Supervision, Writing – review and editing.

## Footnotes

1
https://www.cabidigitallibrary.org/doi/10.1079/cabicompendium.36168

2
https://www.primer3plus.com/

3
https://www.ncbi.nlm.nih.gov/tools/primer-blast/

4
https://www.ncbi.nlm.nih.gov/genbank/

5
https://www.idtdna.com

6
http://www.ncbi.nlm.nih.gov
