# Supplementary material for: Molecular diagnostic assay for pre-harvest detection of Tilletia indica infection in wheat plants
Source: Front Microbiol. 2023 Nov 1;14:1291000. doi: 10.3389/fmicb.2023.1291000 (PMC10646428; doi:10.3389/fmicb.2023.1291000)
Supplement: Supplementary file 1 [file Data_Sheet_1.doc]

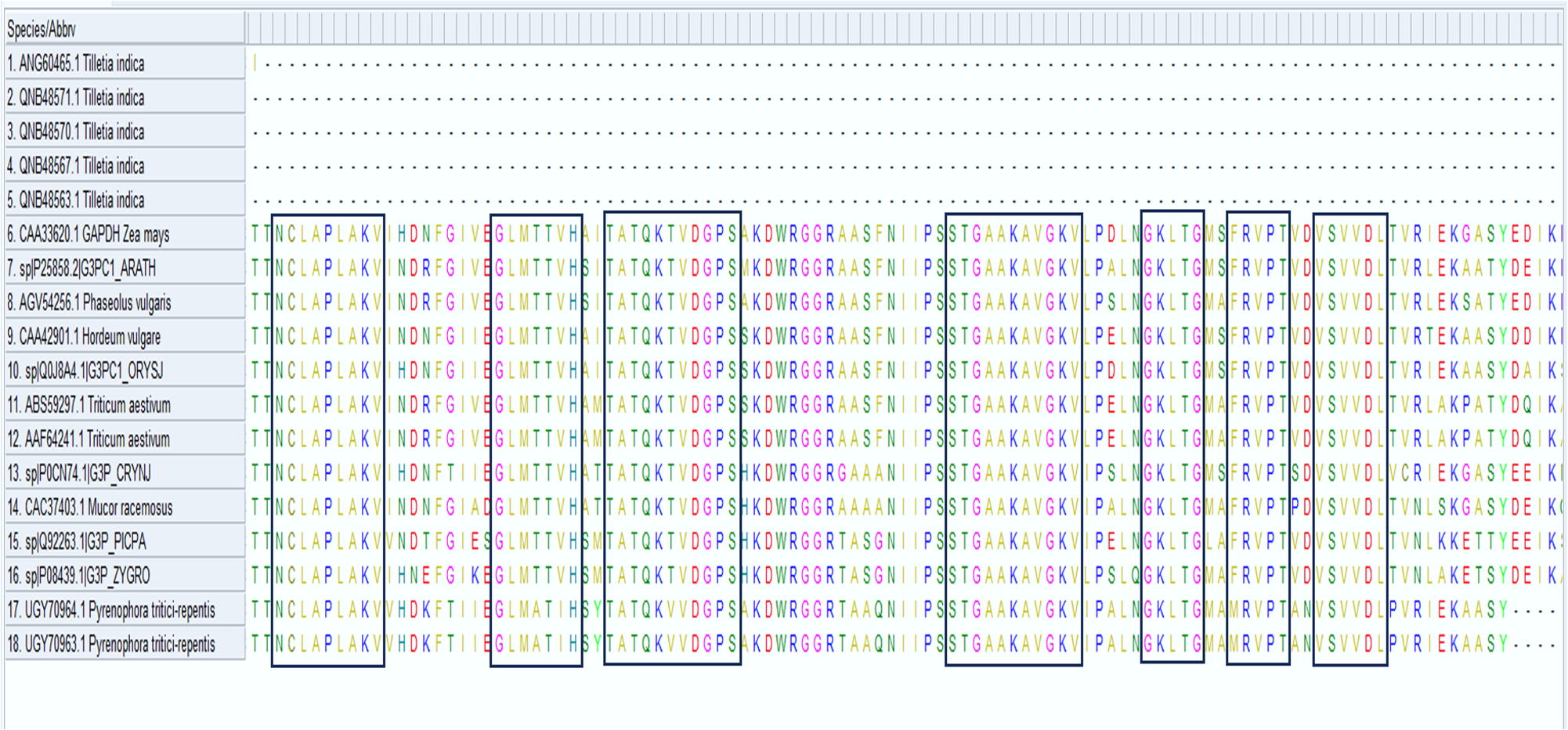


**Fig S1:** Multiple alignment of fungi/animal /plant GAPDH amino acid sequences to indicate the specificity of *T. indica* specific primers
